# Dengue Fever Seroprevalence and Risk Factors, Texas–Mexico Border, 2004

**DOI:** 10.3201/eid1310.061586

**Published:** 2007-10

**Authors:** Joan Marie Brunkard, Jose Luis Robles López, Josue Ramirez, Enrique Cifuentes, Stephen J. Rothenberg, Elizabeth A. Hunsperger, Chester G. Moore, Regina M. Brussolo, Norma A. Villarreal, Brent M. Haddad

**Affiliations:** * University of California, Santa Cruz, California, USA; † Servicios de Salud de la Jurisdicción Sanitaria III, Matamoros, Mexico; ‡ Health Department–City of Brownsville, Brownsville, Texas, USA; § Instituto Nacional de Salud Pública, Cuernavaca, Mexico; ¶ Centro de Investigacíon y de Estudios Avanzados–Instituto Politéchnico Nacional], Mérida, Mexico; # Centers for Disease Control and Prevention, San Juan, Puerto Rico; ** Colorado State University, Fort Collins, Colorado, USA; †† Laboratorio Estatal de Salud Pública de Tamaulipas, Ciudad Victoria, Mexico

**Keywords:** dengue fever, dengue hemorrhagic fever, U.S.–Mexico border, seroprevalence, mosquito-borne disease, poverty, research

## Abstract

Reported autochthonous dengue fever transmission in the United States has been limited to 5 south Texas border counties since 1980. We conducted a cross-sectional serosurvey in Brownsville, Texas, and Matamoros, Tamaulipas, Mexico (n = 600), in 2004 to assess dengue seroprevalence. Recent dengue infection was detected in 2% (95% confidence interval [CI] 0.5%–3.5%) and 7.3% (95% CI 4.3%–10.3%) of residents in Brownsville and Matamoros, respectively. Past infection was detected in 40% (95% CI 34%–45%) of Brownsville residents and 78% (95% CI 74%–83%) of Matamoros residents. For recent infection, only weekly family income <$100 was a significant predictor (adjusted odds ratio 3.2, 95% CI 1.3–8.0). Risk factors that predicted past dengue infection were presence of larval habitat, absence of air-conditioning and street drainage, and weekly family income <$100. Mosquito larvae were present in 30% of households in both cities. Our results show that dengue fever is endemic in this area of the southern Texas–Mexico border**.**

## Abstract 2

High seroprevalence of dengue was found on both sides of the border.

Dengue fever is the most prevalent mosquitoborne viral disease in the world, causing an estimated 50 million infections and 25,000 deaths annually, with at least 2.5 billion persons at risk for transmission (*1*–*4*). Reports of autochthonous dengue fever transmission on the US side of the Texas–Mexico border have been rare—only 64 cases were reported during 1980–1999, compared with 62,514 cases on the Mexican side of the border (*5*–*10*). In the debate over the potential for expansion of dengue and malaria with climate change, the border region has been cited as evidence that mosquitoborne diseases are largely determined by public health capacity and socioeconomic factors, and specifically that US affluence and lifestyle limit transmission of the disease (*5*,*11*–*13*). These conclusions, however, are largely based on incidence reports obtained from passive surveillance that contrast with the epidemiologic dengue situation on the ground.

Recent studies (*14*; J. Brunkard, unpub. data) suggest that dengue is substantially underreported on both sides of the border and prompted us to conduct an epidemiologic investigation in the neighboring cities of Brownsville, Texas, USA, and Matamoros, Tamaulipas, Mexico. Our primary objectives were to assess population seroprevalence of dengue and to identify the most important risk factors for regional transmission. Public health agencies from both countries at the local, state, and national levels collaborated on the project. To our knowledge, this is the first dengue seroprevalence study conducted in the lower Rio Grande Valley since 1980 (*6*).

## Materials and Methods

### Survey Design

In the fall of 2004, we conducted a binational, cross-sectional serosurvey at the household level in Brownsville and Matamoros to measure dengue prevalence in the region. We interviewed members of 300 households in each city for a total sample size of 600. Household selection was probability-based, using a stratified, multistage, cluster-sampling design. In the first stage, 50 census tracts and 50 basic geostatistical areas, the Mexican equivalent of the census tract, were selected by using probability-proportional-to-size sampling with replacement. In the second stage, 3 census blocks were randomly selected from each census tract, and for the final stage, households were randomly or systematically selected.

The sampling frame was based on year 2000 census data for both the United States and Mexico (*15*,*16*). However, at the final stage, we counted all houses in the block on-site and randomly selected starting points, allowing for the incorporation of population changes since the 2000 censuses were conducted.

### Household Serosurvey

We collected a blood sample (5 mL intravenously) from 1 volunteer per household (>15 years of age), conducted larval inspections in and around the house, and interviewed participants by using a household survey that measured risk factors for dengue and public perception about the disease. Two survey teams consisting of 2 interviewers, a medical professional, and an entomologist worked concurrently in both cities to control for seasonal and temporal variance. The survey was timed to coincide with the height of the traditional dengue season (August–December), with most cases occurring in September and October. The survey ran for 5 weeks in October and November 2004. We recorded age, sex, and length of residence in the area for all participants. We attempted to include only those residents who had lived in the region for >10 years, so that our seroprevalence measure would more accurately reflect regional transmission.

Before beginning the study, human subjects approval was obtained from the University of California Institutional Review Board. We pilot tested the survey questionnaire in neighborhoods in both cities in September 2004. Signed, informed consent was given by all survey participants in their preferred language (Spanish or English), including an additional consent form for the Health Insurance Portability and Accountability Act from US survey participants. Participants <18 years of age (n = 6) were required to obtain a parent’s signed consent before giving their own. Participation in the survey was voluntary, and no gifts or financial incentives were offered. Survey participants were notified in person or by mail if they tested positive for recent dengue infection.

### Laboratory Analysis

Serum samples were analyzed at the Laboratorio Estatal de Salud Pública Tamaulipas (State Laboratory in Cd. Victoria, Tamaulipas, Mexico) by using DUO immunoglobulin (Ig) M/IgG capture ELISA (Panbio Inc., Brisbane, Queensland, Australia) to identify recent primary and secondary infections and an indirect IgG ELISA for past dengue infection (Panbio). The Dengue Branch (San Juan, Puerto Rico) of the Centers for Disease Control and Prevention (CDC) conducted confirmatory testing on all samples that tested positive or equivocal for recent infection with capture IgM and IgG ELISAs (Panbio). CDC provided dengue-positive and -negative control serum samples to test on the ELISA kits (Panbio) before testing the serum specimens. CDC also tested a random subsample (n = 12) of serum samples that were negative for recent dengue infection.

### Laboratory-based Classification

Only samples confirmed by CDC were classified as recent infections. CDC criteria included samples with presence of IgM antibodies >0.2 optical density (OD) or presence of IgG antibodies with titers >40,960 (*17*). The IgG ELISA performed by CDC is based on titration of the sera to determine the antibody titer of IgG in the sera. Values of 40,960 are equivalent to the hemagglutination inhibition (HI) titer of 2,560, which the World Health Organization classifies as recent secondary infections (*18*). Past infection was identified by presence of low-titer dengue IgG antibodies, as measured by indirect IgG ELISA (Panbio).

### Plaque Reduction Neutralization Test (PRNT)

Additional confirmatory tests were performed by CDC on 12 positive or equivocal samples by using a 90% reduction in numbers of plaques (PRNT_90_), as previously described (*19*), to determine the specificity of the antibody response to the infecting virus. Samples with a PRNT_90_-positive titer for a single serotype with an IgG titer >10,240 were classified as recent infections.

### Entomologic Survey

We conducted larval sampling in and around the households to identify the mosquito species present and to determine whether the presence of *Aedes aegypti*, the primary dengue vector, was associated with recent or past dengue infection. We surveyed water-holding containers inside and outside the house and collected larvae and pupae. They were identified by entomologists in both city health departments. The data were translated into house and Breteau indices, which are indicators of mosquito vector density (*20*).

### Statistical Analysis

Adjusting the analysis to account for the survey design enables generalization across the statistical population. We used Stata version 9 (Stata Corp., College Station, TX, USA) for all survey design-adjusted descriptive and inferential analyses. We used binomial survey–adjusted Wald tests or Wilcoxon-Mann-Whitney rank sum tests to determine significant differences in frequencies or distributions of key variables, respectively, between Matamoros and Brownsville. We conducted survey design–corrected, multivariate logistic regression based on a multivariate a priori hypothesis. We used the outcomes of recent and past dengue infection as dependent variables in separate models.

Independent variables in all models included *Ae. aegypti* and *Ae. albopictus* mosquito habitat (number of water-holding containers in and around the house), presence of air-conditioning and intact screens, household density, storage of water, street drainage, weekly family income, presence of immature *Ae. aegypti* on the premises, and history of crossing the border within the past 3 months. We constructed 3 models: separate models for recent and past dengue infection and a third model adding a dummy variable for city, which allowed us to identify the independent variables in the model most responsible for the different prevalence in past dengue infection in the 2 cities. Twenty-two exclusions were made because of missing data in the independent variables; all models contained 578 observations. We conducted Fisher exact tests to determine the effect of missing data on the dependent variables. All variables were entered into the model as a block without regard for significance level.

## Results

### Serologic Testing

Serologic evidence of past dengue infection was identified in 40% (95% confidence interval [CI] 34%–45%) of Brownsville residents and 78% (95% CI 74%–83%) of Matamoros residents. An additional 3% of residents in both cities tested equivocal for prior dengue infection. Seroprevalence of IgG dengue antibodies was remarkably consistent with citywide averages across all age groups within both cities except for younger persons (ages 15–24 years) in Brownsville and older persons (ages >65 years) in Matamoros. Seroprevalence was slightly higher in female participants in both cities, but differences were not statistically significant (Table 1).

**Table 1:** Prevalence of IgG dengue antibodies by age and sex, Brownsville, Texas, and Matamoros, Mexico, 2004*

Characteristic	Brownsville, %	Matamoros, %
Age group, y		
15–24	8	79
25–34	45	75
35–44	43	72
45–54	45	80
55–64	35	79
65–74	43	95
>75	38	90
Sex		
Male	35	72
Female	42	80

*IgG, immunoglobulin G.

Following a dengue infection, IgM responses are of limited duration, generally 1–2 months (*21*), and may not be elevated in secondary infections (*22*). Recent dengue infection—as indicated by presence of IgM antibodies >0.2 OD, IgG antibodies >40,960 (*17*), or PRNT_90_ results—was identified in 2% (95% CI 0.5%–3.5%) of Brownsville residents and 7.3% (95% CI 4.3%–10.3%) of Matamoros residents. Most appeared to be secondary infections. Results from the PRNT_90_ assay (n = 3) indicated that dengue serotypes 1 and 2 were circulating in the population (Table 2).

**Table 2:** Serologic test results for serosurvey, Brownsville, Texas, and Matamoros, Mexico, 2004*

Serologic test	Brownsville, n	Matamoros, n
Recent infection†	6	22
IgM >0.2 OD†	1	2
IgG >40,960	5	19
PRNT_90_	1 (DEN-2)	2 (DEN-1)
Past infection‡	119	235

*IgM, immunoglobulin M; OD, optical density; PRNT, plaque reduction neutralization test; DEN, dengue virus. †Laboratory-confirmed by the Dengue Branch, Centers for Disease Control and Prevention, defined by antidengue IgG titer >40,960 or IgM >0.2 OD. ‡Laboratory-confirmed by using indirect IgG ELISA (Panbio Inc., Brisbane, Queensland, Australia).

### Comparison of Panbio Inc. and CDC Test Results

The IgG capture ELISA (Panbio) calculates a positive result based in units. This test determines the sample absorbance compared to a calibrant absorbance. Based on the kit, the interpretation of a positive result is >22 units and the interpretation of this result is suggestive of a recent secondary dengue infection. The CDC IgG ELISA is based on the titration of the antibody present in the serum sample. This titration can be correlated with an HI value to determine a diagnosis of recent secondary dengue infection. When the 2 tests are compared based on the definition of recent secondary dengue infection, the Panbio test is 87.5% sensitive and 100% specific when using the CDC IgG ELISA as the accepted standard. All samples that tested positive for IgG antibodies by Panbio test kits were confirmed by CDC (*3*,*17*).

### Demographics

Mean ages for Brownsville and Matamoros residents were 46.5 and 41.8 years, respectively (range 15–88 years). Most participants were female: 67% in Brownsville and 75% in Matamoros. Based on interviewer observations, we believe that the dominant reason for unequal representation of men in the survey was their reluctance to give blood. There was little difference in mean length of residence in the 2 cities (Brownsville, mean 25.6 years [range 3–77]; Matamoros, mean 29.3 years [range 8–77]). A large percentage of the survey participants had lived in their respective cities their entire lives: 25.3% in Brownsville and 41.7% in Matamoros; 83% of survey participants in Brownsville and 99% in Matamoros had lived in their city >10 years.

### Risk Factors

Many population characteristics were similar between the 2 cities: water and sewerage provision, household size, level of intact screens, and mosquito habitat and density. Key differences (p<0.01) included water storage practices, presence of air-conditioning, street drainage, income, presence of discarded tires, percentage of the population buying drinking water, and travel across the border (Table 3).

**Table 3:** Population characteristics and risk factors for dengue in Brownsville, Texas, and Matamoros, Mexico, 2004

Risk factor	Brownsville, %	Matamoros, %	p value*
Piped water	98	98	1.000
Buy water	95	99.7	<0.001
Sewerage	91	88	0.495
Street drainage	82	48	<0.001
Store water	4	34	<0.001
Screens present	76	64	0.009
Intact screens	40	32	0.054
Air-conditioning (room and central)	83	32	<0.001
Discarded tires	44	20	<0.001
Larval habitat	88	92	0.284
Mosquito larvae present	31	30	0.764
Crossed border (1 mo)	54	38	<0.001
Crossed border (3 mo)	66	45	<0.001
Median household weekly income ($ US)	300	100	<0.001
Mean persons/household	3.9	4.2	0.028
Mean hours/day at home	18.3	19.4	0.022

*Probability values of variables with percentages by adjusted Wald test; the remainder by 2-sample Wilcoxon-Mann-Whitney rank-sum test.

For recent infection, only weekly family income <$100 was significant as a predictor with an adjusted odds ratio (AOR) of 3.2 (95% CI 1.3–8.0), p = 0.01. All other variables were not significant (Table 4). Design effects for all variables included in the model ranged from 0.74 to 1.06, indicating near identical variance to a design using simple random sampling.We ran the same model using past dengue infection as the dependent variable and found several epidemiologic risk factors associated with previous dengue infection: street drainage, air-conditioning, *Ae. aegypti*, and *Ae. albopictus* larval habitat in the neighborhood, and weekly family income <$100 US (Table 5).

**Table 4:** Logistic regression results for recent dengue infection in Brownsville, Texas, and Matamoros, Mexico, 2004*

Variable	Adjusted odds ratio	p value	95% Confidence interval	Deff
Income <$100	3.22	0.012	1.31–7.95	0.95
Missing income	1.35	0.671	0.34–5.42	1.00
Street drainage	0.69	0.395	0.29–1.65	1.00
Larval habitat	2.20	0.381	0.37–13.07	0.74
Air-conditioning	0.74	0.543	0.28–1.96	0.94
Intact screens	0.98	0.959	0.41–2.32	1.06
Store water	1.17	0.709	0.51–2.68	0.90
*Aedes aegypti*	1.05	0.912	0.47–2.31	0.92
Cross border, 3 mo	0.95	0.900	0.40–2.24	1.05
People/household	0.97	0.727	0.80–1.17	0.88

*Missing data in independent variables (n = 22) did not significantly change prevalence of recent or past dengue infection (p>0.10) in the remaining 578 observations used in subsequent models. Deff, design effect, the ratio of variance between the survey design and simple random sampling.

**Table 5:** Logistic regression results for serologic evidence of past dengue infection in Brownsville, Texas, and Matamoros, Mexico, 2004*

Variable	Adjusted odds ratio	p value	95% Confidence interval	Deff
Income <$100	2.59	0.000	1.58–4.26	0.92
Missing income	0.90	0.679	0.54–1.50	0.83
Street drainage	0.57	0.009	0.37–0.87	1.07
Larval habitat	2.35	0.008	1.26–4.41	1.00
Air-conditioning	0.58	0.014	0.38–0.89	1.04
Intact screens	1.35	0.111	0.93–1.95	0.90
Store water	1.62	0.079	0.95–2.76	1.19
*Aedes aegypti*	0.84	0.476	0.53–1.35	1.05
Cross border, 3 mo	0.90	0.581	0.62–1.31	0.93
People/household	1.06	0.300	0.95–1.19	1.31

*Missing data in independent variables (n = 22) did not significantly change prevalence of recent or past dengue infection (p>0.10) in the remaining 578 observations used in subsequent models. Deff, design effect, the ratio of variance between the survey design and simple random sampling.

Past dengue infection was significantly different between the 2 cities (Pearson’s design-based F [1, 98] = 78.01, p<0.0001). We added a city variable to the past infection model to determine its influence in explaining dengue prevalence in our model. In the model, city was highly significant (AOR 4.36, t = 5.74, p<0.0005), and the model F improved from F (10, 89) = 5.42, p<0.0001 to F (11, 88) = 7.14, p<0.0001 with the addition of city to the model. Several variables that predicted past dengue infection changed significantly with the addition of the city variable to the model including stored water, street drainage, air-conditioning, and income, indicating that the influence of these factors on past infection differed by city. We tested for collinearity among all independent variables and found none; variance inflation factors (VIF) for all tests were <1.82, mean VIF = 1.26, far lower than the accepted VIF >10 value for significant collinearity (*23*).

### Entomologic survey

We found mosquito larvae in 30% of households in both cities, but the relative abundance of the species differed between the 2 cities (Table 6). The house index for *Ae. aegypti* differed substantially between the 2 cities (14% and 25% in Brownsville and Matamoros, respectively). *Ae. albopictus*, an exotic species first detected in Texas in the 1980s, was more abundant in Brownsville (13%) than in Matamoros (4%), while *Culex quinquefasciatus* was present at the same level in both cities. Breteau indices for all species were the same as house indices in both cities or differed by <1%.

**Table 6:** House index: percentage of premises positive for a given mosquito species in Brownsville, Texas, and Matamoros, Mexico, 2004

Species	Brownsville, %	Matamoros, %	p value*
*Aedes aegypti*	14	25	0.003
*Ae. albopictus*	13	4	0.0001
*Culex quinquefasciatus*	5	4	0.69

*Probability values by adjusted Wald test.

## Discussion

Brownsville and Matamoros are contiguous cities separated by the Rio Grande (Figure). Of the 6 persons with recent dengue infections in Brownsville, 4 had not crossed the border or traveled outside of the United States in the preceding 3 months and therefore acquired the infections locally (United States). Based on year 2000 census population estimates of 161,546 and 376,279 for Brownsville and Matamoros, respectively, our point prevalence for dengue infections translates to 3,231 undocumented annual dengue infections in Brownsville (95% binomial Wald CI 751–5,711) and 27,581 annual dengue infections in Matamoros (95% binomial Wald CI 16,180–38,757). The dengue season came late in 2004, with several probable cases occurring in Matamoros in December and January after the conclusion of the survey, so our seroincidence rate was likely an underestimate of dengue transmission for that year (*24*).

**Figure:**
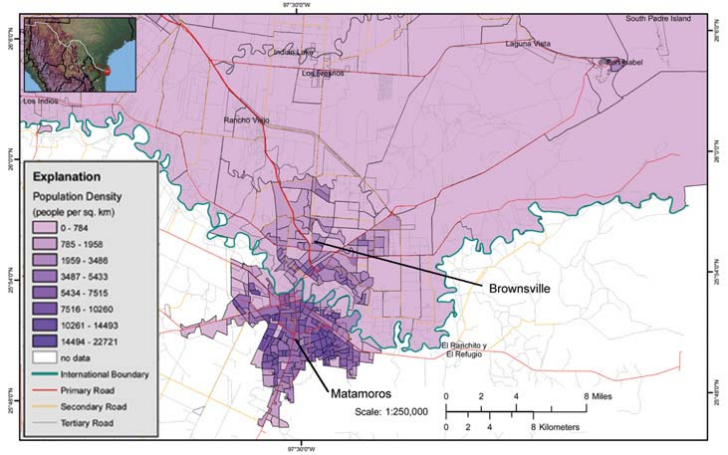
Map of Brownsville, Texas, and Matamoros, Mexico, contiguous cities on the US–Mexico border. Source: US Geological Survey; available from http://borderhealth.cr.usgs.gov/staticmaplib.html

Based on our seroprevalence results for past infection, dengue infections are clearly not being identified by passive surveillance. This result was found in the outbreak of dengue in 1980 in which passive surveillance failed to detect any dengue infections, while Hafkin et al. (*6*) found 63 dengue infections through active surveillance.

Several factors may mask the region’s endemic dengue transmission. One possibility is that the dengue strains circulating in the region result in mostly subclinical infections and mild diseases that do not require hospitalization and are managed through outpatient self-medication such as acetaminophen. Another reason dengue is underreported on the US side of the border may be that a large percentage of these residents cross the border into Mexico for medical diagnoses and treatments. According to our surveys, 59% of Brownsville residents regularly cross the border for medical purposes; however, only 2% of Matamoros residents went to Brownsville for their medical needs. Lack of laboratory resources to confirm dengue infection is another possible explanation. During our survey, physicians in Matamoros reported seeing a large number of patients with suspected dengue, but they were treated with acetaminophen and bed rest because resources were insufficient to conduct laboratory confirmation tests for dengue infection. The most commonly reported illness in the region is the flu.

### Risk Factors

Low income across both cities was the dominant risk factor for both recent and past dengue infection. Poverty is a proxy for many risk factors that make people vulnerable to infectious diseases; some poverty-related factors were measured in this study while others were not. Our specific finding of the protective effect of air-conditioning has been found in another area of the US-Mexico border (*14*). Lack of street drainage appears to limit the ability of mosquito abatement and garbage collection trucks to enter these neighborhoods after a heavy rain. Also, the presence of water-holding containers facilitates vector proliferation in close contact to human hosts.

### Epidemiologic Dynamics

Recent seroepidemiologic studies conducted in dengue-endemic countries have found high dengue seroprevalence: 29.5% in the Brazilian state of Goiás (*25*); 65.7% and 71%, respectively, among schoolchildren in Vietnam (*26*) and Thailand (*27*); 79.5% in Veracruz, Mexico (*28*); and 91% in Managua, Nicaragua (*29*). Historical accounts report widespread outbreaks affecting up to 500,000 people in the US Gulf Coast states during the Galveston, Texas, epidemic in 1922 (*30*) and outbreaks in 1934 and 1941 (*31*). However, very few population-based studies on dengue seroprevalence have been conducted in the United States. The most recent, conducted by Reiter et al. (*14*) in 1999, found 23% seroprevalence in Laredo, Texas, and 48% seroprevalence in Nuevo Laredo, Tamaulipas, Mexico, ≈200 miles (320 km) northwest of our study area. Our population-based study reports the highest seroprevalence of dengue documented in the continental United States since at least 1950.

Demographic factors that could facilitate regional dengue transmission include immigration, which potentially introduces new strains of dengue from dengue-endemic regions in Latin America, and a high local birth rate, which introduces a steady stream of newly susceptible persons. Cocirculation of multiple dengue serotypes has been previously documented in the region (*10*) and suggested from our results by the PRNT_90_, and cases of dengue hemorrhagic fever have increased in Mexico in the past 2 decades (*24*,*32*,*33*). This, coupled with the high background seroprevalence identified in this study, places the border population at greater risk of future dengue hemorrhagic fever outbreaks (*34*,*35*), although the role of sequential infections in disease severity is contested (*36*,*37*).

This study was motivated in part by the climate–dengue debate. While the role of climate change on future dengue transmission is unclear, we find that dengue is already a problem in this area of the US–Mexico border. Because dengue infections are not being identified through local surveillance efforts, we recommend proactive physician outreach emphasizing the potential for dengue infections and increased access to dengue diagnostic tests, especially on the Mexican side of the border, where a large proportion of US and Mexican border residents seek their primary medical care. Improved systems of active binational surveillance for dengue infections are needed, and sentinel sites should include the network of high-volume private clinicians practicing at the border. Ultimately, investments in local infrastructure, improvements in household screening, economic assistance for air-conditioning in dengue-endemic areas, and sustained community education about the importance of reducing larval habitat around the home will be necessary to reduce dengue transmission in this region.

## References

[1] Monath TP. Dengue: the risk to developed and developing countries. Proc Natl Acad Sci U S A. 1994;91:2395–400. 10.1073/pnas.91.7.2395 8146129 PMC43378

[2] Gubler DJ. Dengue and dengue hemorrhagic fever. Clin Microbiol Rev. 1998;11:480–96. 9665979 10.1128/cmr.11.3.480 PMC88892

[3] World Health Organization. Dengue haemorrhagic fever: diagnosis, treatment, prevention, and control. Geneva: The Organization; 1997.

[4] Gibbons RV, Vaughn DW. Dengue: an escalating problem. BMJ. 2002;324:1563–6. 10.1136/bmj.324.7353.1563 12089096 PMC1123504

[5] Reiter P. Climate change and mosquito-borne disease. Environ Health Perspect. 2001;109(Suppl 1):141–61. 10.2307/3434853 11250812 PMC1240549

[6] Hafkin B, Kaplan JE, Reed C, Elliott LB, Fontaine R, Sather GE, Reintroduction of dengue fever into the continental United States. I. Dengue surveillance in Texas, 1980. Am J Trop Med Hyg. 1982;31:1222–8. 7149106 10.4269/ajtmh.1982.31.1222

[7] Centers for Disease Control and Prevention. Underdiagnosis of dengue—Laredo, Texas, 1999. MMWR Morb Mortal Wkly Rep. 2001;50:57–9. 11243446

[8] Rawlings JA, Hendricks KA, Burgess CR, Campman RM, Clark GG, Tabony LJ, Dengue surveillance in Texas, 1995. Am J Trop Med Hyg. 1998;59:95–9. 9684635 10.4269/ajtmh.1998.59.95

[9] Centers for Disease Control and Prevention. Imported and indigenous dengue fever—United States, 1986. MMWR Morb Mortal Wkly Rep. 1987;36:551–4. 3112547

[10] Centers for Disease Control and Prevention. Dengue fever at the U.S.-Mexico border, 1995–1996. MMWR Morb Mortal Wkly Rep. 1996;45:841–4. 8927003

[11] Intergovernmental Panel on Climate Change (IPCC). Climate change 2001: impacts, adaptation and vulnerability. Technical summary. Geneva: IPCC Secretariat; 2001.

[12] US National Research Council, Committee on Climate Ecosystems Infectious Disease and Human Health. Under the weather: climate, ecosystems, and infectious disease. Washington: National Academy Press; 2001.

[13] Gubler DJ, Reiter P, Ebi KL, Yap W, Nasci R, Patz JA. Climate variability and change in the United States: potential impacts on vector- and rodent-borne diseases. Environ Health Perspect. 2001;109(Suppl 2):223–33. 10.2307/3435012 11359689 PMC1240669

[14] Reiter P, Lathrop S, Bunning M, Biggerstaff B, Singer D, Tiwari T, Texas lifestyle limits transmission of dengue virus. Emerg Infect Dis. 2003;9:86–9. 12533286 10.3201/eid0901.020220 PMC2873752

[15] US Census Bureau. Census 2000 summary file 1, 2000. [cited 2004 Sep 9]. Available from http://factfinder.census.gov/servlet/DTGeoSearchByListServlet?ds_name=DEC_2000_SF1_U&_lang=en&_ts=185398395501

[16] Instituto Nacional de Estadística Geografía e Informática (INEGI). XII Censo general de población y vivienda, 2000 [cited 2004 Sep 9]. Available from http://www.inegi.gob.mx/est/contenidos/espanol/proyectos/censos/cpv2000/bd/pv2000/metadatos/poblaciontotal.asp?c=6176

[17] Miagostovich MP, Nogueira RM, dos Santos FB, Schatzmayr HG, Araujo ES, Vorndam V. Evaluation of an IgG enzyme-linked immunosorbent assay for dengue diagnosis. J Clin Virol. 1999;14:183–9. 10.1016/S1386-6532(99)00059-1 10614855

[18] World Health Organization (WHO). Dengue haemorrhagic fever: diagnosis, treatment, and control. Albany (NY): WHO Publications Center USA; 1986.

[19] Russell PK, Nisalak A, Sukhavachana P, Vivona S. A plaque reduction test for dengue virus neutralizing antibodies. J Immunol. 1967;99:285–90. 6031202

[20] Service MW. Mosquito ecology: field sampling methods. London: Elsevier Applied Science; 1993.

[21] Innis BL, Nisalak A, Nimmannitya S, Kusalerdchariya S, Chongswasdi V, Suntayakorn S, An enzyme-linked immunosorbent assay to characterize dengue infections where dengue and Japanese encephalitis co-circulate. Am J Trop Med Hyg. 1989;40:418–27. 2540664 10.4269/ajtmh.1989.40.418

[22] Ruechusatsawat K, Morita K, Tanaka M, Vongcheree S, Rojanasuphot S, Warachit P, Daily observation of antibody levels among dengue patients detected by enzyme-linked immunosorbent assay (ELISA). Jpn J Trop Med Hyg. 1994;22:9–12.

[23] Chatterjee S, Hadi AS, Price B. Regression analysis by example. New York: Wiley; 2000.

[24] Secretariat of Health. Mexico. Boletin Epidemiologia. 2005. [cited 26 Nov 2005]. Available from http://www.dgepi.salud.gob.mx/boletin/boletin.htm

[25] Siqueira JB, Martelli CM, Maciel IJ, Oliveira RM, Ribeiro MG, Amorim FP, Household survey of dengue infection in central Brazil: spatial point pattern analysis and risk factors assessment. Am J Trop Med Hyg. 2004;71:646–51. 15569799

[26] Thai KT, Binh TQ, Giao PT, Phuong HL, Hung le Q, Van Nam N, et al. Seroprevalence of dengue antibodies, annual incidence and risk factors among children in southern Vietnam. Trop Med Int Health. 2005;10:379–86. 10.1111/j.1365-3156.2005.01388.x 15807802

[27] Tuntaprasart W, Barbazan P, Nitatpattana N, Rongsriyam Y, Yoksan S, Gonzalez JP. Seroepidemiological survey among schoolchildren during the 2000–2001 dengue outbreak of Ratchaburi Province, Thailand. Southeast Asian J Trop Med Public Health. 2003;34:564–8. 15115129

[28] Navarrete-Espinosa J, Acevedo-Vales JA, Huerta-Hernandez E, Torres-Barranca J, Gavaldon-Rosas DG. [Prevalence of dengue and leptospira antibodies in the state of Veracruz, Mexico] [in Spanish]. Salud Publica Mex. 2006;48:220–8. 10.1590/S0036-36342006000300006 16813130

[29] Balmaseda A, Hammond SN, Tellez Y, Imhoff L, Rodriguez Y, Saborio SI, High seroprevalence of antibodies against dengue virus in a prospective study of schoolchildren in Managua, Nicaragua. Trop Med Int Health. 2006;11:935–42. 10.1111/j.1365-3156.2006.01641.x 16772016

[30] Rice L. Dengue fever: preliminary report of an epidemic at Galveston. Tex State J Med. 1922;189:217–8.

[31] Ehrenkranz NJ, Ventura AK, Cuadrado RR, Pond WL, Porter JE. Pandemic dengue in Caribbean countries and the southern United States—past, present and potential problems. N Engl J Med. 1971;285:1460–9. 4941592 10.1056/NEJM197112232852606

[32] Briseño-García B, Gόmez-Dantés H, Argott-Ramírez E, Montesano R, Vázquez-Martínez AL, Ibáñez-Bernal S, et al. Potential risk of dengue hemorrhagic fever: the isolation of serotype dengue 3 in Mexico. Emerg Infect Dis. 1996;2:133–5. 10.3201/eid0202.960210 8903215 PMC2639818

[33] Navarrete-Espinosa J, Gómez-Dantés H, Germán Celis-Quintal J, Vázquez-Martínez JL. Clinical profile of dengue hemorrhagic fever cases in Mexico. Salud Publica Mex. 2005;47:193–200. 10.1590/S0036-36342005000300002 16104461

[34] Halstead SB. Observations related to pathogensis of dengue hemorrhagic fever. VI. Hypotheses and discussion. Yale J Biol Med. 1970;42:350–62. 5419208 PMC2591710

[35] Halstead SB, Rojanasuphot S, Sangkawibha N. Original antigenic sin in dengue. Am J Trop Med Hyg. 1983;32:154–6. 6824120 10.4269/ajtmh.1983.32.154

[36] Rosen L. The Emperor's New Clothes revisited, or reflections on the pathogenesis of dengue hemorrhagic fever. Am J Trop Med Hyg. 1977;26:337–43. 869095 10.4269/ajtmh.1977.26.337

[37] Murgue B, Deparis X, Chungue E, Cassar O, Roche C. Dengue: an evaluation of dengue severity in French Polynesia based on an analysis of 403 laboratory-confirmed cases. Trop Med Int Health. 1999;4:765–73. 10.1046/j.1365-3156.1999.00478.x 10588771

